# Nursery Aid Society and Medical Relief

**Published:** 1899-01-28

**Authors:** 


### Editor's Letter-Box.

[Our Correspondents are reminded that prolixity is a great bar to publication, and that brevity of style and conciseness of statement greatly facilitate early insertion.]

### NURSERY AID SOCIETY AND MEDICAL RELIEF.

Dr. W. Ledingham Christie writes: I notice in an article on medical relief some recommendations about self-supporting dispensaries and nursing organisations for the poor. I found several years ago, when at a children's hospital, the vast need for some means of getting a knowledge of infant management and dieting into the actual homes of the people. I find no method so successful and beneficial as self-supporting dispensaries attached to a small cottage hospital and nursing home. Ladies attached to and helping in the support of the home make clothes and help to provide milk and medical comforts for the poor, especially the infants. I would recommend that such institutions be started in every district, and that special teaching be given so that mothers, particularly, should be instructed and nurses sent out free to the poor and at a small fee to others, so that infantile mortality may be reduced. I feel sure that over 30,000 infants, born healthy, are annually sacrificed to ignorance alone.

